# Dynamic changes in gut microbiota during pregnancy among Chinese women and influencing factors: A prospective cohort study

**DOI:** 10.3389/fmicb.2023.1114228

**Published:** 2023-03-29

**Authors:** Muxia Li, Guohua Zhang, Lijun Cui, Lin Zhang, Qian Zhou, Chenxue Mu, Ruixin Chi, Na Zhang, Guansheng Ma

**Affiliations:** ^1^Department of Nutrition and Food Hygiene, School of Public Health, Peking University, Beijing, China; ^2^The Third Department of Obstetrics, Shijiazhuang Obstetrics and Gynecology Hospital, Shijiazhuang, China; ^3^The Seventh Department of Obstetrics, Shijiazhuang Obstetrics and Gynecology Hospital, Shijiazhuang, China; ^4^Department of Pediatrics, The Third Hospital of Hebei Medical University, Shijiazhuang, China; ^5^Department of Computer Science, City University of Hong Kong, Hong Kong, Hong Kong SAR, China; ^6^Laboratory of Toxicological Research and Risk Assessment for Food Safety, Peking University, Beijing, China

**Keywords:** pregnancy, gut microbiota, dynamic changes, gestational diabetes mellitus, metagenomics analysis

## Abstract

Gut microbiota (GM) dynamics during pregnancy vary among different populations and are affected by many factors, such as living environments and diet. This study aims to observe and evaluate the changes in the structure and function of the GM from the first to the third trimester of pregnancy in Chinese women, and to explore the main factors affecting the changes in intestinal microecology. Fifty-five Chinese pregnant women were recruited for this study and their fecal samples were collected during the first (P1), second (P2), and third trimesters (P3) of pregnancy. We exploited metagenomic sequencing to compare the composition and function of the GM in different pregnancy periods. Bioinformatic analysis revealed that there were differences in the composition of the GM among P1, P2, and P3, as indicated by the increase in α-diversity and β-diversity of the GM and the differences in the relative abundances of distinct bacterial phyla. Gestational diabetes mellitus (GDM) was the main factor (*P* < 0.05) that affected the changes in GM at various stages of pregnancy. There were also disparities in the structure of the GM between the GDM group and non-GDM group in the P1, P2, and P3. The GDM group exhibited increased abundances in *Ruminococcus_gnavus*, *Akkermansia_muciniphila*, *Alistipes_shahii*, *Blautia_obeum*, and *Roseburia_intestinalis;* while, the abundances of *Bacteroides coprocola*, *Bacteroides plebeius*, *Erysipelatoclostridium ramosum*, and *Prevotella copri* were increased in the non-GDM group. Three of the four species enriched in the non-GDM group manifestied significantly negative correlations with the insulin-signaling pathway and lipopolysaccharide biosynthesis (*r* ≤ −0.3, adjusted *P* < 0.05). In the GDM group, *Bacteroides vulgatus* and *Ruminococcus gnavus* were significantly and positively correlated with insulin signaling pathway and lipopolysaccharide biosynthesis (*r* ≤ −0.3, adjusted *P* < 0.05) among the species enriched from early pregnancy. Virtually all of the species enriched in P2 and P3 were positively correlated with steroid hormone biosynthesis. These results suggest a potential role for the GM in the development of GDM, enabling the potential prevention of GDM by targeting the GM.

## Introduction

Pregnancy is an extra-ordinary biologic process that women may experience during their lives, which involves a variety of physiologic changes. Some of these changes have been demonstrated, such as alterations in hormonal and metabolic levels. Only in recent years, investigators have gradually appreciated that the gut microbiota (GM) dynamics during pregnancy may also participate in physiologic activities and further regulate metabolism, thereby affecting the health of both mother and offspring (Nuriel-Ohayon et al., 2016).

Several reports observed have revealed attenuated microbial diversity of the GM accordance with pregnancy progression throughout the three trimesters (Koren et al., 2012; DiGiulio et al., 2015; Cortez et al., 2019; Wu et al., 2020). During early pregnancy, the composition of the GM resembles that of healthy non-pregnant individuals (Koren et al., 2012; Cortez et al., 2019). However, the composition of the GM gradually changes with the progression of pregnancy; that is, a diminution in α-diversity (the diversity of species within individuals) and commensurate elevation in β-diversity (a dissimilarity between individuals). In the third trimester of pregnancy, the relative abundance of Proteobacteria and Actinobacteria with their pro-inflammatory effects increase significantly, becoming the dominant microbiota (Ponzo et al., 2019a). In contrast, the relative abundances of butyrate-producing bacteria, such as *Clostridium* and *Eubacterium* decreased (Koren et al., 2012; DiGiulio et al., 2015; Wu et al., 2020). These results suggest that the GM may be involved in mediating glucose and lipid metabolism and inflammatory responses during pregnancy, and that these metabolic changes modulate intestinal microecology throughout pregnancy and even postpartum, thus constituting one of the ways by which the health of offspring is affected. However, the mechanism underlying this action remains unclear. A previous study showed that obese pregnant women had reduced abundances of intestinal *Bifidobacteria* and *Bacteroides*, and significantly increased abundances of *Staphylococcus* and *Escherichia coli*, compared with normal weight women during pregnancy (Santacruz et al., 2010; Gomez-Arango et al., 2016). Gestational weight gain (GWG) can reduce the diversity of the GM during pregnancy, increase the abundance of *E. coli*, and decrease the number of *Bifidobacterium* and *Akk* bacteria (Stanislawski et al., 2017). GM dynamics were also altered for pregnant women with pregnancy complications such as gestational diabetes mellitus (GDM) and hypertension (Kuang et al., 2017; Liu et al., 2017; Chen et al., 2020). Studies have shown that the GM may be a key factor affecting the occurrence and development of GDM. The α-diversity of the GM in GDM patients was significantly reduced, and the content of *Parobacteria* and *Klebsiella* was higher, while the abundances of *Bifidobacterium* and *Eubacterium* were higher in healthy pregnant women (Kuang et al., 2017), indicating that the GDM state is related to the GM imbalance. In addition, the abundances of Actinobacteria, *Collinsella* and *Desulfovibrio* in the gut of the GDM women were elevated relative to that of euglycemic pregnant women, and this difference persisted until after delivery. The diversity of the GM was also reduced in patients with preeclampsia, with the numbers of pathogenic bacteria such as *Clostridium perfringens* and *Breedella* increasing and the numbers of beneficial bacteria such as *Coprococcus dextris* decreasing as the pregnancy progressed (Liu et al., 2017; Stanislawski et al., 2017), this indicated the GM of patients with preeclampsia contained a certain degree of pathogenicity and was involved in the procession of this disease. In brief, GM plays an important role in the occurrence and development of various pregnancy-related diseases by participating in various metabolic pathways, and in immune regulation and inflammation in the human body. In addition, the association between maternal GM and spontaneous preterm birth, gestational hypothyroidism, postpartum depression and other factors also needs to be explored by supplemental studies.

Intriguingly, the GM can be orchestrated by various factors such as host genetics and lifestyle (Rothschild et al., 2018; Redondo-Useros et al., 2020). Investigators have recently ascertained that environments imposed dominant effects on the GM (He et al., 2018; Sun et al., 2021), suggesting differential GM dynamics during pregnancy in distinct populations. Therefore, exploration of GM variations during pregnancy in diverse populations should improve GM-targeted interventions (Deschasaux et al., 2018; Rothschild et al., 2018).

There are few extent reports on gestational variations in the GM for Chinese women in particular environments and who have adopted special dietary styles. In this study, we conducted a prospective cohort study to evaluate the related factors affecting GM the dynamics in pregnant women in Shijiazhuang, Hebei Province. The aim of this study was to evaluate the changes in the GM structure and function from early to late pregnancy and to discern the main factors that affected the changes in intestinal microecology in this specific geographic region and wit respect to specific dietary style. Our study should provide novel evidence for the presence fo the GM dynamics during pregnancy, based on findings from our specific population.

We hypothesize that (1) there are differences in the composition of the GM among the first (P1), second (P2), and third trimesters (P3) of pregnancy. (2) nutrient intake or health status comprise the main factors affecting the changes in the GM. (3) there are also differences in the composition and function of the GM with respect to the primary affecting factors.

## Materials and methods

### Study design

This was a prospective cohort study, and recruitment was conducted at Shijiazhuang Obstetrics and Gynecology Hospital, Hebei Province, China. Sixty-four pregnant women who met the inclusion and exclusion were recruited. Inclusion criteria were women (1) aged between 18 and 35 years; (2) with established medical records and who underwent regular prenatal examination since early pregnancy (11–13 weeks of gestation) at the Shijiazhuang Obstetrics and Gynecology Hospital; (3) of Han nationality; (4) who experienced natural conception; and (5) who were willing to be followed up until the third trimester (35–37 weeks of gestation) of pregnancy. Exclusion criteria were women (1) with infectious diseases such as AIDS, active hepatitis, syphilis, etc.; (2) with chronic diseases such as cardiovascular disease, kidney disease, digestive diseases and other diseases before pregnancy; (3) undergoing multiple pregnancies; and (4) who used antibiotic and/or microbiologic agents within the previous month. During the follow-up, nine pregnant women were lost to follow-up due to early miscarriage or other personal reasons. Ultimately, 55 pregnant women were included in this study.

This study was conducted in Shijiazhuang City, Hebei Province, which is located in northern China. The environment in this area is a temperate monsoon climate, and diets chiefly consist of cereals and vegetables. Dietary intake by pregnant women during P1, P2, and P3 was measured using a validated semi-quantitative food frequency questionnaire (FFQ), and food intake was recorded for the month prior to each visit. The questionnaire listed 119 foods, in which the type, frequency, and amount of food were assessed. Nutrient intake was assessed by trained researchers using the *Chinese Food Composition Table*.

Pregnancy complications and other disorders and symptoms were diagnosed by obstetricians according to their respective diagnostic criteria (Rouse et al., 2016; Mack and Tomich, 2017; Sinkey et al., 2020), in combination with the participant’s clinical presentation, physical examination, and corresponding laboratory tests.

### Ethical review

The study protocol was reviewed and approved by the Peking University Biomedical Ethics Committee (IR0001052-19150), and conducted in accordance with the principles of the Declaration of Helsinki. All participants read the informed consent forms before enrollment, voluntarily agreed to participate in this study, and signed the informed consent forms. Signed informed consent (in duplicate) was obtained from each participant prior to participation in this study, with one provided to the participant and the other to the researchers.

### Data collection

The participants were asked to fill in a study questionnaire requesting the participants’ basic demographic details. And anthropometric measurements were taken by trained nursing staff; measurements included height, weight and blood pressure. Body mass index (BMI) was calculated as weight (kg)/height (cm)^2^. The participants were asked to fill in the FFQ and provided their fecal samples during P1, P2, and P3 of pregnancy. A total of 5 g of fecal sample was collected by each participant in the morning with a clean sterile spoon and placement into sterile and airtight tube on the day of each visit, and this was stored at −80°C within two hours after collection.

### DNA extraction and sequencing

Microbial DNA was extracted following the protocol included in the QIAamp DNA stool kit (Qiagen). The quality and quantity of the extracted DNA were measured using NanoDrop (Thermo Scientific) and Qubit (Thermo Fisher Scientific, Singapore). The DNA fragment size was evaluated by agarose gel electrophoresis. Then, the short-insert DNA libraries were generated using a NEBNext^®^ UltraTM DNA Library Prep Kit (NEB, USA), and we executed 2 × 150bp paired-end sequencing via an Illumina NovaSeq 6000 platform (Illumina, San Diego, CA, United States).

### Metagenomic analysis

Raw sequencing reads were filtered to trim low-quality sequences, library primers and adaptors using Trimmomatic (v0.39.2) with default parameters (Bolger et al., 2014). The host DNA sequences were then removed by mapping filtered reads against the human genome database (version hg38) employing bowtie2 (v2.3.5.1) (Langmead and Salzberg, 2012). The unmapped high-quality reads were annotated via MetaPhlAn3 (v3.0.13) (Beghini et al., 2021). We constructed the gene functional profiles by with Humann3 against the built-in marker gene database, and the abundances of taxonomic profiles and KEGG pathways were then calculated by read counts (Beghini et al., 2021). The indices of microbial diversity were assessed by applying the Qiime2 package (Bolyen et al., 2019).

### Statistical and comparative metagenomic analysis

All statistical analyses were performed in R (version 3.4.1). We selected for further analysis those genera and species that represented ≥ 0.01% abundance in at least one subject. Principal coordinate analysis (PCoA) based on the *vegan* package was adopted to reduce data dimensions. The impact of various factors on GM distributions was assessed with permutational multivariate analysis of variance (PERMANOVA) with 9,999 permutations (package *vegan* in R) (Oksanen et al., 2013), and Bray-Curtis distance was applied to assess GM dissimilarity between samples using the *adonis2* function (in the *vegan* package in R). Shannon diversity, Bray-Curtis dissimilarity, and the abundances of microbiota were compared using the Wilcoxon rank sum test (inter-group) or the Kruskal–Wallis test (among three groups). Spearman correlation was executed to evaluate the association between microbial features and gene functions (as visualized by the *pheatmap* package in R) (Kolde and Kolde, 2018). The *p*-values for multiple tests were adjusted with the Benjamini–Hochberg method using the “function p.adjust.” Adjusted *p*-value <0.05 were defined as statistically significant. Data visualization was conducted with the *ggplot2* package in R (Wickham, 2016).

## Data availability

The raw data were submitted to the CNGB Sequence Archive (CNSA) under Project no. CNP0003669. The sample-related metadata in this article are available upon reasonable request.

## Results

### Characteristics of subject recruitment

**FIGURE 1:**
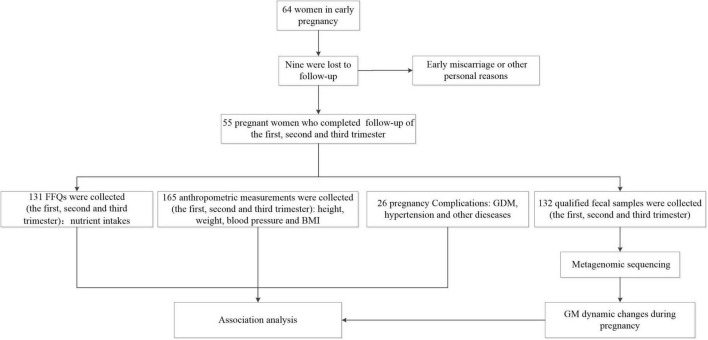
Procedures regarding of basic information and sample collection in the present study.

A total of 55 Chinese women were analyzed, and their characteristics were depicted in Table 1 and Supplementary Table 1. The majority of the recruited pregnant women in our study exhibited a normal BMI (*n* = 33/55, 60%). There were also 13 overweight (23.6%), and nine obese (16.4%). Two recruited individuals manifested pre-pregnancy diabetes and hypertension (at enrollment, the condition was stable, with blood glucose and blood pressure in the normal ranges). Of all subjects, 14 (*n* = 14/55, 25.5%) were diagnosed with GDM, nine (*n* = 9/55, 16.4%) with gestational hypertension or preeclampsia, and nine (*n* = 9/55, 16.4%) with anemia. The collection process was displayed in Figure 1.

**TABLE 1:** Basic characteristic of study subjects.

	All (*N* = 55)
**Age**	
Mean ± SD	30.07 ± 4.01
**Parity, *n* (%)**	
First birth	33 (60)
Second birth	22 (40)
**Educational status, *n* (%)**	
Primary	2 (3.6)
Secondary	24 (43.7)
Tertiary	29 (52.7)
**Monthly income (yuan), *n* (%)**	
<5,500	26 (47.3)
5,500–10,000	17 (30.9)
>10,000	3 (5.5)
Unknown	9 (16.4)
**Pre-pregnancy weight (kg)**	
Mean ± SD	62.35 ± 11.64
**Pre-pregnancy BMI (kg/m^2^)**	
Mean ± SD	23.70 ± 4.31
Normal	33 (60.0)
Overweight	13 (23.6)
Obese	9 (16.4)
**Pre-pregnancy diabetes, *n* (%)**	
No	54 (98.2)
Yes	1 (1.8)
**Pre-pregnancy hypertension, *n* (%)**	
No	54 (98.2)
Yes	1 (1.8)
**Use of folic acid, *n* (%)**	
Not taking	4 (7.3)
3 months before menopause	13 (23.6)
2 months before menopause	9 (16.4)
After menopause	29 (52.7)
**Smoking status, *n* (%)**	
Never	53 (96.4)
Occasionally	2 (3.6)
**Drinking status, *n* (%)**	
Never	48 (87.3)
Occasionally	7 (12.7)
**Pregnancy complications, *n* (%)**	
GDM	14 (25.5)
Gestational hypertension/preeclampsia	9 (16.4)
Anemia	9 (16.4)

### Microbiotal composition and gene profiles

After data filtration, the total number of clean reads was 11.29 Gb (37.33 ± 13.92 Mb, median ± SD). The ratio of taxonomic annotation averaged 58.95% based on the MetaPhlAn database, and the mapped gene number averaged 217,577 based on the HUMAnN database.

### GM differences among the first, second and third trimesters

**FIGURE 2:**
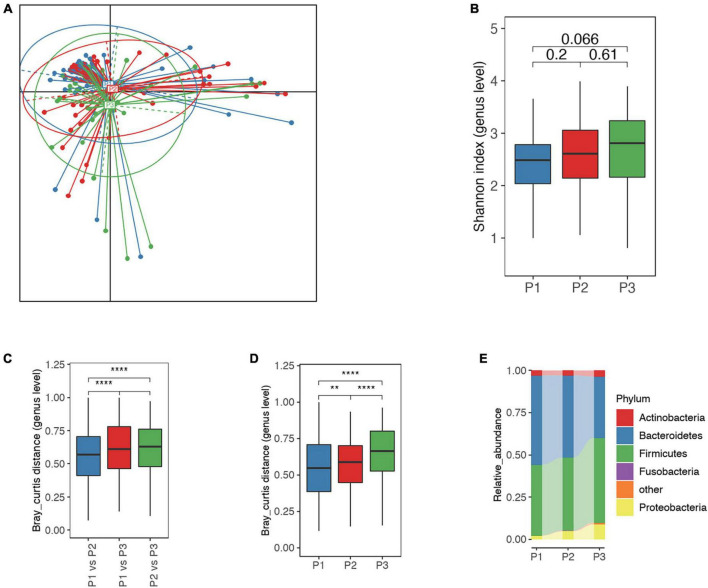
Overall differences in the gut microbiota (GM) among P1, P2 and P3. **(A)** Principal coordinate analysis (PCoA) based on Jensen-Shannon divergence as to distance measurement, suggested that microbial distribution in P3 samples differed from those in P1 and P2. **(B)** Comparison of alpha-diversity for P1, P2, and P3 microbial samples; the number represents adjusted *P*-value. **(C)** The Bray-Curtis distance for microbial samples between P1 and P2 was significantly different from the distance between P1 and P3 as well as between P2 and P3, suggesting greater variation in the GM in P3. **(D)** Inter-individual Bray-Curtis distance of microbial samples at each time point, indicating greater heterogeneity in microbial samples in P3. **(E)** Phylum-level microbial structure at P1, P2, and P3 timepoints. Bacteroidetes continued to decreased and Proteobacteria to increase commensurate with trimester. P1, P2, and P3 represent the first, second and third trimesters, respectively. The Wilcoxon rank-sum test was applied to analyze the significance of inter-group differences, and the *P*-value was adjusted with the Benjamini–Hochberg method. **, **** represent adjusted *P* < 0.01 and *P* < 0.0001, respectively.

Principal coordinate analysis indicated that microbial samples were distributed according to sampling timepoints P1, P2, and P3 (Figure 2A). In consistence, we found increased microbial diversity as the trimester progressed, though this was no statistical significant (Figure 2B). Further analysis showed that the dissimilarity in microbial samples between the P1 and P2 was lower than that between P3 and P1 or P2, though this was also not statistically significant (Figures 2A, C). Additionally, intra-group variations increased notably and phylum-level GM structure changed following trimester progression (Figures 2D, E). For instance, the level of Bacteroidetes decreased (averaging 52.85, 48.66, and 36.33% in P1, P2, and P3, respectively) and Proteobacteria elevated (averaging 2.07, 4.83, and 8.89% in P1, P2, and P3, respectively) as trimesters progressed (Figure 2E).

### GDM contributes significantly to inter-individual variations in GM dynamics

**FIGURE 3:**
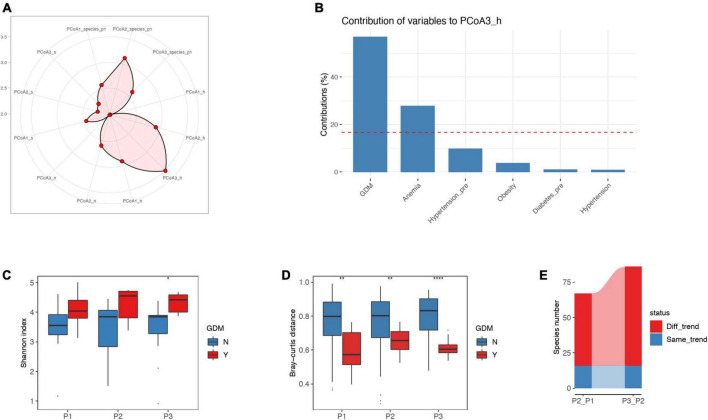
Factors impacting GM dynamics in accordance with trimester progression. **(A)** Permutational multivariate analysis of variance (PERMANOVA) was adopted to assess the effects of several indices on GM dynamics: the variable was more significant when the point was located nearer the outer circle. PCoA1_species_p1, PCoA2_species_p1, and PCoA3_species_p1 represent the first, second, and third principal components for baseline species-level GM structures, respectively. PCoA1_h, PCoA2_h, and PCoA3_h represent the first, second, and third principal components for health status, including GDM, hypertension, anemia, obesity, pre-pregnancy diabetes and hypertension. PCoA1_n, PCoA2_n, and PCoA3_n represent the first, second, and third principal components for daily nutrient intake at each trimester, including micro- and macro-nutrients. PCoA1_s, PCoA2_s, and PCoA3_s represent the first, second, and third principal components for socioeconomic status, including income and education. Analytic result exhibited a notable association between PCoA3_h and GM dynamics. **(B)** Contribution of factors in PCoA3_h, suggesting that GDM contributed ≥ 50% to the PCoA3_h. **(C)** The differences in Shannon index between GDM and non-GDM women at the P1, P2, and P3 timepoints. Women with GDM manifested higher microbial diversity than non-GDM women, and the difference was significant at the P3 timepoints. **(D)** Inter-individual Bray-Curtis distance with respect to microbial samples for women with GDM was significantly lower than that for non-GDM women, suggesting a greater similarity of microbial samples in the GDM group. **(E)** For the P2_P1 GM changes, 16 species possessed similar changing trends (increase or decrease) and 51 species showed different changing trends between GDM and non-GDM women. For the P3_P2 GM changes, 16 species had a similarly changing trend and 70 species had different changing trends between GDM and non-GDM women. P1, P2, and P3 represent the first, second, and third trimesters, respectively. The Wilcoxon rank-sum test was applied to analyze the significance of inter-group differences, and the *P*-value was adjusted by the Benjamini–Hochberg method. *, **, **** represent adjusted *P* < 0.05, *P* < 0.01 and *P* < 0.0001, respectively.

Spearman correlation analysis showed that there was no significant correlation between nutrient intake and gut microbiota (top 20 species level bacteria) during pregnancy (Supplementary Table 2). In our analysis, we included factors that may affect GM dynamics, such as baseline mid-level GM structure, nutrient intake (including macronutrients and micronutrients), health status, and socioeconomic status. Permutational multivariate analysis of variance (PERMANOVA) indicated that the third principal component of health status (PCoA3_h) contributed significantly to the time-associated GM variations (adjusted *P* < 0.05) (Figure 3A). And we also determined that GDM represented the most important component for PCoA3_h, suggesting a notable association between the GDM and GM dynamics (Figure 3B). Further analysis showed that microbial diversity in the GDM group was higher than that in the non-GDM group (adjusted *P* < 0.05 in the P3) (Figure 3C). Additionally, the inter-individual dissimilarity of microbial samples in the GDM group was markedly lower than that in the non-GDM group, suggestive of higher GM structural homogeneity in the GDM group, and the significance was higher in the P3 (Figure 3D). We also consistently found increased numbers of species showing a changing trend (increase or decrease) between the GDM and non-GDM as the trimester progressed (Figure 3E).

### Differences in structural and functional GM dynamics between women with GDM and those without GDM

**FIGURE 4:**
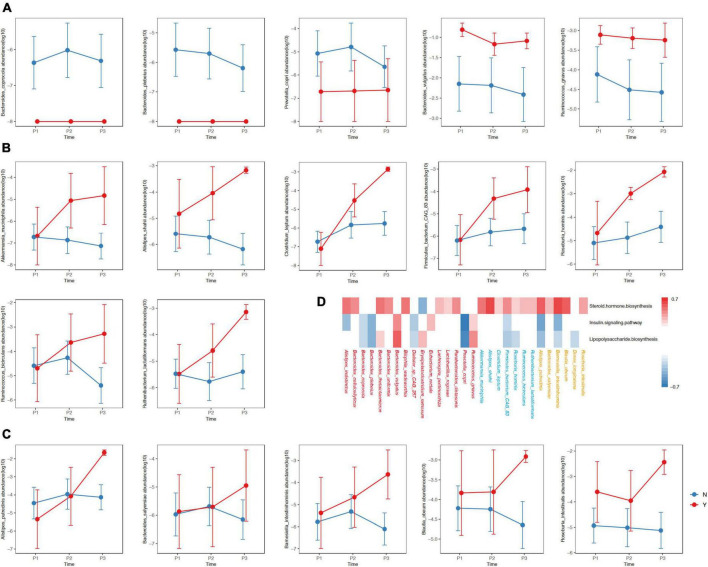
Differences in the relative abundances of species between women with of without GDM at the three timepoints. The y-axis in Panels **(A–C)** represent log10 values of the relative abundances. The blue and red lines represent non-GDM and GDM groups, respectively. **(D)** The red, blue and orange colors of the species designate differing distributions among groups with respect to P1, P2, and P3, respectively. The colors in the heatmap reflect positive (red, *r* ≥ 0.3, adjusted *P* < 0.05) and negative (blue, *r* ≤ –0.3, adjusted *P* < 0.05) associations between the kegg3 function and the selected species. P1, P2, and P3 indicate the first, second, and third trimesters, respectively. The *P*-value was adjusted using the Benjamini–Hochberg method.

We observed three types of differential dynamics with regard to microbial species that began to accumulate in GDM or non-GDM women at P1, P2 and P3 (Figures 4A–C). For the species showing different abundances between GDM and non-GDM groups at each trimester, four continued to accumulate in the non-GDM group from P1 onward: *Bacteroides coprocola*, *Bacteroides plebeius*, *Erysipelatoclostridium ramosum* and *Prevotella copri* (Figure 4A). Further analysis indicated that three of these four species possessed a notably negative correlation with the insulin-signaling pathway and lipopolysaccharide biosynthesis (*r* ≤ −0.3, adjusted *P* < 0.05), but we noted no significant correlation with steroid hormone biosynthesis (Figure 3D). Of the species enriched in the GDM group at the three sampling timepoints, *Bacteroides vulgatus*, and *Ruminococcus gnavus* were noteworthy in their positive association with the insulin-signaling pathway as well as lipopolysaccharide biosynthesis (*r* ≤ −0.3, adjusted *P<*0.05) (Figures 4A, D). For species that accumulated in the GDM group from P2 onward, seven were positively associated with steroid hormone biosynthesis, while only one species (*Firmicutes_bacterium_CAG_83*) was slightly negatively associated with the insulin-signaling and lipopolysaccharide biosynthesis (Figures 4A, D). For species enriched in the GDM group at the P3 onward, five were positively associated with steroid hormone biosynthesis, including *Alistipes putridinis* and *Barnesiella intestinihominis;* and also associated negatively with insulin signaling or lipopolysaccharide synthesis (Figures 4A, D).

## Discussion

Emerging studies have revealed the presence of GM dynamics during pregnancy (Koren et al., 2012; DiGiulio et al., 2015; Nuriel-Ohayon et al., 2016, Neuman and Koren, 2017, Cortez et al., 2019). As GM structures can be modulated by host and environmental factors, it is important to explore gestational GM dynamics for different populations and then apply personalized GM-targeted interventions to improve health during pregnancy.

Consistent with prior reports (Koren et al., 2012; DiGiulio et al., 2015; Ferrocino et al., 2018; Abdullah et al., 2022), we observed gradual GM changes that paralleled the progression of pregnancy. We herein found a gradual increase in the α-diversity index (Shannon index) during P1, P2 and P3 of pregnancy, as reported in a previous Italian study (Ferrocino et al., 2018). In contrast, other reports found no obvious changes in GM diversity during pregnancy (DiGiulio et al., 2015; Abdullah et al., 2022). This discrepancy may be partially explained by different health statuses, diet and lifestyles specific to distinct populations (He et al., 2018; Rothschild et al., 2018; Redondo-Useros et al., 2020; Sun et al., 2021). Congruent with previous studies, we identified increased β-diversity of the GM in normal pregnancies (Koren et al., 2012; Ferrocino et al., 2018). Compositional GM changes were also reported during pregnancies, including increased Proteobacteria and decreased Bacteroides as trimesters progressed (Ponzo et al., 2019a), and this was consistent with our findings. Increased Proteobacteria may reduce glucose homeostasis, insulin resistance and low inflammatory responses in pregnant women (Koren et al., 2012; Ponzo et al., 2019a), suggesting an association between gestational changes in GM and health status.

In the current study, we observed that GDM was the most significant contributor to GM dynamics when compared to hypertension, age, diet or other factors; this was in accordance with various reports that indicated disproportionate GM structures in pregnant women with GDM (Karlsson et al., 2013; Le Chatelier et al., 2013; Crusell et al., 2018; Ferrocino et al., 2018; Wu et al., 2020; Zheng et al., 2020; Mokkala et al., 2021; Abdullah et al., 2022). Women with GDM tend to exhibit reduced insulin sensitivity and increased inflammation, which are generally related to reduced diversity of GM (Le Chatelier et al., 2013). Previous studies (Wu et al., 2020; Ferrocino et al., 2018; Abdullah et al., 2022) have also shown a relatively lower tendency for GM α-diversity indices (Ace, Chao, Shannon, and Simpson) in pregnant women with GDM compared to women without GDM. In addition, some studies failed to report any differences in α-diversity between GDM and non-GDM groups in late pregnancy (Crusell et al., 2018; Zheng et al., 2020). We speculate that these inconsistent findings were caused by specifically individualized GM, which can be modulated by host genetics and lifestyle (Rothschild et al., 2018; Redondo-Useros et al., 2020). Some studies (Barrett et al., 2018; Ponzo et al., 2019a) showed that dietary composition during pregnancy was an important factor affecting GM during pregnancy. However, we did not find any significant correlation between nutrient intake and GM in our sdury, and nutritional composition was not the primary factor affecting the dynamic changes of GM; this may be relate to the fact that our participants were all from the same region and had similar dietary structure and habits. In the future, larger-sample and multi-center studies are needed to explore the correlation between dietary intake and GM in Chinese pregnant women and its impact on the dynamic changes inherent to GM.

Our results also implied that there were differences in the composition of and trends in changes between the GDM group and the non-GDM group as the pregnancy progressed; this was in accordance with various reports that indicated inordinate GM structures in pregnant women with GDM (Kuang et al., 2017; Crusell et al., 2018; Ferrocino et al., 2018; Wang et al., 2018; Cortez et al., 2019; Ponzo et al., 2019b; Liu et al., 2020; Zheng et al., 2020). Several scientific groups have reported increases in the Firmicutes/Bacteroidetes ratio, *Blautia, Roche, Bilophila*, and *Bifidobacterium* among women with GDM compared to those without GDM (Ferrocino et al., 2018; Ponzo et al., 2019b; Zheng et al., 2020), as well as increases in *Collinsella, Megamonas*, and *Doreas* among GDM women in the P3 (Kuang et al., 2017; Crusell et al., 2018; Cortez et al., 2019). However, the abundances of short-chain fatty acid (SCFA)-producing bacteria, such as *Faecalibacterium, Ruminococcus, Roseburia, Coprococcus, Akkermansia, Phascolarctobacterium*, and *Eubacterium* were reduced (Kuang et al., 2017; Crusell et al., 2018; Wang et al., 2018; Cortez et al., 2019; Liu et al., 2020). In the resent study, we noted that the relative abundance of *P.copri* decreased continuously throughout pregnancy in the GDM group; and that *Parabacteroides_distasonis, Lactobacillus_rogosae, Bacteroides_thetaiotaomicron, Bacteroides_uniformis, R.gnavus and B.vulgatus* were enriched from early pregnancy onward. *Firmicutes_bacterium_CAG_83* and *Ruthenibacterium_lactatiformans* were enriched from P2 onward, while *Blautia_obeum* and *Dorea_longicatena* increased in P3. We herein also found that some bacterial species, such as *Akkermansia_muciniphila, Ruminococcus_bicirculans, Roseburia_hominis*, and *Clostridium_leptum* accumulated in women with GDM, while other studies showed diminutions in women with GDM (Kuang et al., 2017; Liu et al., 2020; Wang et al., 2020; Wu et al., 2020; Xu et al., 2020). These distinctions emphasize the differential findings with respect to GM dynamics in GDM, and the necessity for the adoption of personalized GM-targeted interventions in different populations.

Other reports further revealed a role for the forementioned GM components in GDM. In a Chinese population-based study (Li et al., 2021), *R.gnavus* was found to be associated with elevated fasting blood glucose. It was also reported that *R.gnavus* could synthesize and secrete glucorhamnan, a complex polysaccharide with a rhamnose skeleton and glucose side chain, and that it effectively induced dendritic cells to secrete inflammatory cytokines (such as tumor necrosis factor, TNF-α) (Henke et al., 2019). We recognize that inflammation can impair glucose homeostasis, thereby altering insulin signaling (Mokkala et al., 2017), and we observed a correlation between GDM group-enriched GM components and the insulin-signaling pathway. For example, *R.gnavus* was found to be enriched in P1 of pregnancy and positively correlated with the insulin-signaling pathway. This finding may support the concept that increased *R.gnavus* in early pregnancy is associated with the development of GDM. In addition, GDM group-enriched *B.vulgatus* (among the microorganisms enriched in early pregnancy) also showed a significant positive correlation with the insulin-signaling pathway.

We identified a marked association between *R. gnavus* and *B. vulgatus* with the biosynthesis of lipopolysaccharide (LPS), an endotoxin that can induce low-grade inflammation and insulin resistance (Rosadini and Kagan, 2017; Cardona et al., 2018; Huang et al., 2021). The LPS contained in Bacteroidetes is a strong activator of Toll-like receptor 4 (TLR4), and the combination of LPS and TLR4 can activate a wide range of cell-signaling pathways, thereby inducing inflammatory responses and the expression and secretion of cytokines (Medzhitov and Horng, 2009). LPS biosynthesis is also positively correlated with blood glucose at the individual level (Kuang et al., 2017). In contrast, *P.copri* (which was more abundant in non-GDM women) was significantly negatively correlated with LPS biosynthesis.

Although our study encompasses changes in the GM during the natural course of pregnancy and the chief factors affecting these changes, and provides potential microbial markers for GDM monitoring, there were some limitations. (1) The single-center design is not extrapolable to the situation in different regional populations of China and (2) the small sample size of the present study, which contained only 55 participants, may lead to some bias in the analysis. Therefore, more work is needed in future studies. We posit (1) conducting large multicenter cohort studies to confirm our findings; (2) validating microbial markers for different geographic regions and ethnic groups; and (3) further analyzing the gut microbial functional pathways associated with GDM.

In conclusion, we observed variations in intestinal microecology from P1 to P3 of pregnancy, particular in P3, and noted that GDM was the primary factor affecting the changes in gut microbiota during pregnancy. Moreover, in women with and without GDM, the specific composition of GM varied at different pregnancy periods and associated with the insulin-signaling pathway as well as LPS biosynthesis, suggesting a potential role for GM in GDM development while providing potential GM-targeted prevention of GDM. This study could provide an additional reference for understanding the dynamics of gestational GM in disparate populations.

## Data availability statement

The data presented in this study are deposited in the CNGB (https://db.cngb.org/search/project/CNP0003669/) repository, accession number: CNP0003669.

## Ethics statement

The studies involving human participants were reviewed and approved by the Peking University Biomedical Ethics Committee (IR0001052-19150). The patients/participants provided their written informed consent to participate in this study.

## Author contributions

GM managed the project. QZ and LC recruited the participants. ML, CM, and RC performed the sampling and information collection. LZ and QZ performed the bioinformatics analysis and guided the statistic analysis in this work. ML interpreted the analysis results and wrote the manuscript. NZ and GM supervised and polished the manuscript. All authors reviewed the manuscript.
