# Supplementary material for: Dynamic changes in gut microbiota during pregnancy among Chinese women and influencing factors: A prospective cohort study
Source: Front Microbiol. 2023 Mar 29;14:1114228. doi: 10.3389/fmicb.2023.1114228 (PMC10096036; doi:10.3389/fmicb.2023.1114228)
Supplement: Supplementary file 1 [file Table_1.DOCX]

**Table S1** Detail information of the participants

|  | P1 | P2 | P3 | *p*-value |
| --- | --- | --- | --- | --- |
| Anthropometrics indicators |  |  |  |  |
| Weight^b^ | 65.74±12.78 | 71.71±10.95 | 76.58±10.33 | 0.018^*^ |
| BMI^b^ | 24.84±5.21 | 27.26±4.67 | 28.93±4.32 | 0.036^*^ |
| SBP (mmHg) | 113.26±14.43 | 115.79±14.95 | 122.63±21.59 | 0.234 |
| DBP (mmHg)^bc^ | 68.74±14.60 | 71.37±12.34 | 81.00±13.72 | 0.019^*^ |
| Nutrition intake |  |  |  |  |
| Daily energy intake (kcal) | 1334.57 ±707.01 | 1695.51±1094.61 | 1835.80±1044.71 | 0.264 |
| Daily protein intake (g) | 70.63 (48.89-115.81) | 63.89 (49.44-92.79) | 64.78 (40.49-94.91) | 0.822 |
| Daily fat intake (g) | 53.57±31.49 | 70.69±50.13 | 73.27±46.44 | 0.323 |
| Daily carbohydrate intake (g) | 180.21±90.73 | 208.13±135.15 | 226.13±129.88 | 0.499 |
| Daily dietary fiber intake (g) | 13.99±9.48 | 14.15±12.77 | 16.31±11.08 | 0.775 |
| Daily cholesterol intake (mg) | 402.10 (220.26-509.40) | 388.60 (302.09-735.89) | 458.15 (300.10-57.73) | 0.508 |
| Daily vitamin A intake (µgRAE)^ab^ | 89.10 (38.62-339.98) | 529.90 (222.45-902.65) | 606.00 (286.22-875.66) | 0.005^*^ |
| Daily riboflavin intake (mg)^abc^ | 0.50 (0.27-0.73) | 25.12 (12.57-53.76) | 1.46 (0.83-3.21) | <0.001^*^ |
| Daily vitamin C intake (mg)^ab^ | 40.31 (21.82-110.80) | 92.35 (60.20-196.07) | 91.58 (66.36-142.54) | 0.033^*^ |
| Daily vitamin E intake (mg)^b^ | 322.65±283.53 | 416.68±133.94 | 590.56±360.86 | 0.014^*^ |
| Daily folic acid intake (mg) | 554.62±386.97 | 681.98±280.94 | 810.49±322.95 | 0.073 |
| Daily calcium intake (mg) | 179.20 (121.23-373.50) | 227.34 (157.19-355.89) | 245.04 (183.41-379.57) | 0.481 |
| Daily iron intake (mg)^bc^ | 12.77 (8.16-20.05) | 18.71 (15.44-23.15) | 35.32 (25.20-59.86) | <0.001^*^ |
| Daily zinc intake (mg)^bc^ | 16.99±8.98 | 24.45±11.38 | 37.56±15.15 | <0.001^*^ |
| Daily selenium intake (mg) | 40.08±26.32 | 60.02±51.96 | 54.76±29.84 | 0.250 |
| Daily iodine intake (µg) | 60.75 (33.80-81.27) | 78.83 (43.79-148.76) | 64.91 (39.71-203.32) | 0.203 |
| Daily fatty acid intake(g) | 43.04 (15.00-68.27) | 40.73 (23.19-58.09) | 51.53 (38.28-94.73) | 0.188 |
| Daily SFA intake (g) | 8.31 (5.21-8.31) | 15.47 (9.39-22.17) | 17.61 (9.78-23.61) | 0.063 |
| Daily MUFA intake (g) | 5.03 (2.56-11.13) | 9.45 (4.87-15.36) | 8.66 (6.52-17.64) | 0.106 |
| Daily PUFA intake (g)^b^ | 13.43±12.45 | 20.85±17.03 | 25.89±14.53 | 0.040^*^ |
| Biochemical indicators |  |  |  |  |
| OGTT response 0h | - | 4.789±0.373 | - |  |
| OGTT response 1h | - | 7.563±0.981 | - |  |
| OGTT response 2h | - | 6.332±0.906 | - |  |

*Normal distributed continuous variables were presented as mean* ± *standard deviation and analyzed using one-way ANOVA, and pairwise comparisons were compared by LSD-t test; non-normal distributed continuous variables were presented as medians and interquartile ranges (Q1-Q3) and analyzed using the Kruskal-Wallis H test. ^*^indicates significant differences between groups; ^a^ indicates significant difference between P1 and P2; ^b^ indicates significant difference between P1 and P3; ^c^ indicates significant difference between P2 and P3.*

*P1, first trimester; P2, second trimester; P3, third trimester; BMI, body mass index; SBP, systolic blood pressure; DBP, diastolic blood pressure; SFA, saturated fatty acid; MUFA, monounsaturated fatty acid; PUFA, polyunsaturated fatty acid; OGTT, oral glucose tolerance test.*
